# Association between Carotid Intima-Media Thickness and the Use of Biological or Small Molecule Therapies in Patients with Rheumatoid Arthritis

**DOI:** 10.3390/diagnostics12010064

**Published:** 2021-12-28

**Authors:** Marta Rojas-Giménez, Clementina López-Medina, Jerusalem Calvo-Gutiérrez, María Ángeles Puche-Larrubia, Ignacio Gómez-García, Pedro Seguí-Azpilcueta, María del Carmen Ábalos-Aguilera, Desirée Ruíz, Eduardo Collantes-Estévez, Alejandro Escudero-Contreras

**Affiliations:** 1 Rheumatology Department, Reina Sofía University Hospital, Maimonides Institute for Biomedical Research of Córdoba (IMIBIC), University of Córdoba (UCO), 14004 Cordoba, Spain; rojasgimenezm@gmail.com (M.R.-G.); yeru83@gmail.com (J.C.-G.); mangeles.puche@gmail.com (M.Á.P.-L.); ignaciogomgar@gmail.com (I.G.-G.); desiree.ruiz@hotmail.com (D.R.); 2 Reina Sofia University Hospital, Maimonides Research Institute of Biomedical Medicine from Cordoba (IMIBIC), University of Córdoba, 14004 Cordoba, Spain; pedromsegui@telefonica.net (P.S.-A.); mc.abalos@outlook.com (M.d.C.Á.-A.); Educollantes@yahoo.es (E.C.-E.); alexcudero2@gmail.com (A.E.-C.)

**Keywords:** rheumatoid arthritis, subclinical atherosclerosis, cardiovascular risk, biological therapy

## Abstract

Objective: The objective of this study was to assess the association of carotid intima-media thickness (CIMT), and also the presence of atheromatous plaque, with biological and targeted synthetic disease-modifying antirheumatic drugs, in an established cohort of patients with rheumatoid arthritis (RA). Patients and Methods: We conducted a cross-sectional observational study based on a cohort of patients with RA and a registry of healthy controls, in whom the CIMT and presence of atheromatous plaque were assessed by ultrasound. Data were collected on disease activity, lab results and treatments. Descriptive and bivariate analyses were performed and two multivariate linear regression models (with CIMT as the dependent variable) were constructed to identify variables independently associated with CIMT in our sample of patients with RA. Results: A total of 176 individuals (146 patients with RA and 30 controls) were included. A higher percentage of patients than controls had atheromatous plaque (33.8% vs. 12.5%, *p* = 0.036), but no differences were found in terms of CIMT (0.64 vs. 0.61, *p* = 0.444). Compared to values in patients on other therapies, the CIMT was smaller among patients on tumour necrosis factor alpha (TNFα) inhibitors (mean [SD]: 0.58 [0.10] vs. 0.65 [0.19]; *p* = 0.013) and among those on Janus kinase inhibitors (mean [SD]: 0.52 [0.02] vs. 0.64 [0.18]; *p* < 0.001), while no differences were found as a function of the use of the other therapies considered. The multivariate linear regression analysis to identify factors associated with CIMT in our patients, adjusting for traditional cardiovascular risk factors such as hypertension, high levels of low-density lipoproteins, diabetes mellitus and smoking, showed that male sex, older age and having a greater cumulative erythrocyte sedimentation rate were independently associated with a larger CIMT, while patients on TNFα inhibitors had a CIMT 0.075 mm smaller than those on other treatments. Conclusions: The use of TNFα inhibitors may protect against subclinical atherosclerosis in patients with RA, patients on this biologic having smaller CIMTs than patients on other disease-modifying antirheumatic drugs. Nonetheless, these results should be confirmed in prospective studies with larger sample sizes.

## 1. Introduction

Patients with rheumatoid arthritis (RA) have an elevated cardiovascular risk, associated with higher mortality rates and a relative risk of a cardiovascular event of approximately 2 compared to individuals of the same age and sex without the disease [1,2,3]. Between a third and half of premature deaths in patients with RA are due to cardiovascular disease (including ischaemic heart disease and stroke) [2,4,5]. This is attributable to accelerated atherogenesis [6], which is influenced by both traditional and non-traditional cardiovascular risk factors (CVRFs), including systemic inflammation [3,7,8,9,10].

For this reason, the early detection of cardiovascular disease is essential in these patients. Assessment of the carotid intima-media thickness (CIMT) and carotid atheromatous plaque using ultrasound has been described as a useful approach for detecting subclinical atherosclerosis [11,12,13]. Even in the absence of atheromatous plaque, CIMT is a marker of high cardiovascular risk and a significant predictor of the development of atheromatous plaque [14].

Some research has suggested that inflammation plays a role in the process of atherosclerosis, through an increase in the expression of proinflammatory cytokines and adhesion molecules (such as the tumour necrosis factor alpha [TNFα] and interleukin 6 [IL-6]) [15]. These may be involved in various stages of atherosclerosis from endothelial dysfunction to the development of atheromatous plaque, and make individuals more vulnerable, the process of atherosclerosis being much more than a mere accumulation of lipids in arterial walls [16,17]. All this implies that proper control of the inflammatory process in these patients should be one of the main goals in the management of atherosclerosis [18].

Nonetheless, few studies have investigated the effect of anti-rheumatic treatments, that help to control inflammation, on CIMT in patients with RA. Among studies that have investigated this issue, a few have assessed the effects of methotrexate, antimalarial drugs and other conventional synthetic disease-modifying antirheumatic drugs (DMARDs) such as leflunomide and sulfasalazine [19,20] and we have found one assessing the effect of biological therapies (including etanercept, infliximab, adalimumab and rituximab) [21]. Overall, there is a paucity of data, and moreover, studies to date have not included the targeted therapies currently available for managing the disease, such as Janus kinase inhibitors (jakinibs). Therefore, the main objective of this study was to assess the association of CIMT, as well as the presence of atheromatous plaque, with biological DMARDs (bDMARDs) and targeted synthetic DMARDs (tsDMARDs), also known as small molecule inhibitors, in an established cohort of patients with RA.

## 2. Patients and Methods

### 2.1. Study Design and Population 

We conducted a cross-sectional observational study based on a cohort of patients with RA and a registry of healthy controls. The study was carried out in the Department of Rheumatology, at Reina Sofia University Hospital (HURS) and the Maimonides Biomedical Research Institute of Cordoba (IMIBIC). All participants gave written informed consent to their inclusion in the study.

### 2.2. Study Population and Protocol

We recruited consecutive patients with a diagnosis of RA according to the 2010 ACR/EULAR criteria through specialist clinics [22]. We excluded patients with any other concomitant inflammatory rheumatic disease or an active infection as well as any who were pregnant. We also included a control group, composed of people with no diagnosis of any inflammatory rheumatic disease, recruited consecutively from among individuals accompanying patients to their appointments. All participants underwent the following procedures: the collection of a fasting blood sample, a physical examination following a set protocol and a carotid ultrasound scan. The study was approved by the Clinical Research Ethics Committee of Reina Sofia University Hospital (reference number: 1217-N-21).

### 2.3. Variables

The main outcome variable was CIMT as measured by B-mode ultrasound, performed by a single expert radiologist, using a Philips EPIQ-7 system and a broadband linear array transducer (5–14 MHz). Using this ultrasound, as well as measuring CIMT, we assessed the presence of atheromatous plaque, defined by consensus [23,24] as focal thickening of the arterial wall encroaching into the lumen at least 0.5 mm or at least 50% of the surrounding CIMT, or a CIMT of greater than 1.5 mm.

We collected data on the following lab results: levels of total cholesterol, triglycerides, low- and high-density lipoprotein cholesterol, homocysteine (normal range 0.68–1.62 mg/L), glucose, and apolipoprotein (Apo) B (normal 65–130 mg/dL) and A1 (normal 105–220 mg/dL), rheumatoid factor (RF) (positive if >14 UI/mL), anti-cyclic citrullinated peptide (anti-CCP) (positive if >7 UI/mL), and C-reactive protein (CRP) (normal <10 mg/dL), as well as the ApoB/ApoA1 ratio and erythrocyte sedimentation rate (ESR) (normal <20 mm/h). ESR and CRP level were considered both at the time of the study visit and in terms of cumulative ESR and CRP, retrospectively, calculating the mean CPR level and ESR over all the blood tests performed at the hospital in the previous 24 months.

We assessed patient inflammatory activity using the cumulative scores on the 28-joint Disease Activity Score with ESR (DAS28-ESR), Simplified Disease Activity Index (SDAI) and Clinical Disease Activity Index (CDAI); calculated as the mean of two annual measurements taken in the previous 24 months. We also included variables related to severity such as the presence of radiological erosions as rated by a trained observer, and the Assessment Questionnaire (HAQ) score assessed at the time of the study visit.

We recorded all the treatments with conventional synthetic DMARDs, tsDMARDs and/or bDMARDs patients were receiving at the time of the study visit and the duration of these treatments. Further, we documented treatments with statins and/or glucocorticoids received at the time of the visit.

### 2.4. Statistical Analysis

First, a descriptive analysis was performed. Qualitative data were expressed as absolute numbers and percentages and quantitative data as means and standard deviations. Chi-square tests were used for comparing categorical variables and Student’s *t* test for independent samples for comparing the means of continuous variables provided that the data were normally distributed. Baseline characteristics, comorbidities and lab results were compared between the study groups (patients with RA and controls).

Subsequently, linear correlation analysis was performed (calculating Pearson’s correlation coefficient) to assess the relationship between CIMT and various patient clinical characteristics and lab results. Patients were classified according to whether they were receiving treatment with specific bDMARDs or tsDMARDs (TNFα inhibitors, IL-6 inhibitors, rituximab, abatacept or jakinibs; for each yes/no) and bivariate analyses were carried out to compare CIMT and the presence of atheromatous plaque between patients who were and were not receiving each of these types of therapy.

Finally, two multivariate linear regression models (one including TNF inhibitors and other including jakinibs as explanatory variable) (DV: CIMT) were constructed to determine the variables that were independently associated with CIMT in the sample of patients with RA. Variables found to be significant in the bivariate analysis and those considered clinically relevant were selected for this analysis.

For all the analysis, *p* values ≤ 0.05 were considered significant and 95% confidence intervals were estimated. We used the R 2.4–0 statistical programme.

## 3. Results

### 3.1. Demographic, Laboratory and Disease-Related Data 

We included a total of 176 individuals (146 patients with RA and 30 controls). Table 1 summarises the baseline demographic and epidemiological characteristics, lab results, comorbidities and carotid ultrasound findings in each group. Most of the patients were women, and overall, their mean age was 55 years. Patients had a higher body mass index (BMI) than controls (*p* = 0.014) and were more likely to have dyslipidaemia (*p* = 0.004). At the time of the study, statins were being taken by 33 out of the 46 patients with dyslipidaemia but none of the controls (*p* = 0.003).

Regarding blood test results, patients with RA had significantly higher levels of homocysteine and higher CRP than controls, with no significant differences in lipid levels. In terms of the characteristics of the disease, the majority of patients were RF and anti-CCP positive, and a third of them had erosions. More than half of the patients were in remission or had low disease activity (DAS28 ≤ 3.2) at the time of the study visit and 58.1% of them were on corticoids, receiving a mean (SD) of 3.7 mg (4.2) of prednisone or equivalent.

Regarding other treatments, more than half of the patients were being treated with biologics, TNFα or IL-6 inhibitors in most cases. Notably, 21 (81%) of the patients on TNFα inhibitors received them in combination with MTX, while 13 (59.1%) of the patients on IL-6 inhibitors were on monotherapy and the others (40.9%) were concomitantly treated with another DMARD. The mean treatment durations (SD) with TNFα and IL-6 inhibitors were 2.6 (2.9) and 1.9 years (2.1), respectively. Among the patients not treated with a biologic (*n* = 70), MTX was the most commonly used drug.

On carotid ultrasound, a higher percentage of patients than controls were found to have atheromatous plaque (33.8% vs. 12.5%, *p* = 0.036), but no differences were observed in terms of CIMT (0.64 vs. 0.61, *p* = 0.444). Analysing the patients who were not on statins (*n* = 113), CIMT values were comparable to those of controls. Further, CIMT values were similar in patients who were and were not on statins (mean, 0.65 vs. 0.62 mm; *p* = 0.373), but fewer of the patients on statins had atheromatous plaque (*n*, 15 vs. 30; *p* = 0.045).

### 3.2. Relationship of CIMT with Disease Characteristics and Lab Results

In the patients, CIMT was observed to be positively correlated with levels of uric acid (r = 0.273, *p* = 0.008), apoB (r = 0.250, *p* = 0.006), total cholesterol (r = 0.253, *p* = 0.003), triglycerides (r = 0.217, *p* = 0.014) and homocysteine (r = 0.363, *p*= 0.003), BMI (r = 0.202, *p* = 0.021), and above all, age, this variable showing the strongest association (r = 0.656, *p* < 0.001). No correlations were found with disease activity indices (DAS28, CDAI, or SDAI) or with RF or anti-CCP. Regarding inflammatory reactants, we observed a weak correlation between CIMT and cumulative ESR (r = 0.183; *p* = 0.032), but no correlation with ESR measured at the time of the visit (r= 0.068; *p* = 0.442) or with CPR either at the time of the visit or the cumulative value. No relationship was observed between treatment duration with biologics and mean CIMT (r = −0.219; *p* = 0.081) at the time of the study visit.

### 3.3. Comparison between Different Biological Therapies

Subsequently, carotid ultrasound findings were compared as a function of the biological or targeted therapies used. CIMT was smaller in patients treated with bDMARDs or tsDMARDs than those on conventional DMARDs alone (mean (SD): 0.60 (0.11) vs. 0.67 (0.23); *p* = 0.019), no such difference being found in the case of atheromatous plaque (*n* (%): 25 (32.9) vs. 24 (34.3); *p* = 0.554). Among the different biological therapies, patients on TNFα inhibitors had a smaller CIMT than those on other treatments (mean (SD): 0.58 (0.10) vs. 0.65 (0.19); *p* = 0.013), and similarly, CIMT was smaller in patients on jakinibs than those on other treatments (mean (SD): 0.52 (0.02) vs. 0.64 (0.18); *p* < 0.001), while no differences were found as a function of the use of the other therapies considered (Table 2). On the other hand, patients on rituximab or abatacept patients were more likely than those not treated with these biologics to have atheromatous plaque.

Comparing the characteristics of patients on TNFα inhibitors with those on other treatments, the only differences found in terms of cardiovascular risk factors were that the former had lower rates of hypertension and lower levels of homocysteine (Supplementary Table S1). We did not observe differences between the groups in the use of glucocorticoids (17 patients on TNFα inhibitors (65.4) vs. 66 (55) not on TNFα inhibitors; *p* = 0.354) or statins (6 patients on TNFα inhibitors (23.07) vs. 29 (24.7) not on TNFα inhibitors; *p* = 0.906). Further, comparing patients on jakinibs with those on other therapies, we found no significant differences between groups in terms of CVRFs, glucocorticoid use (5 patients on jakinibs (62.5) vs. 78 (56.5) not on jakinibs; *p* = 0.757], or statin use (2 patients on jakinibs (25) vs. 33 (23.9) not on jakinibs; *p* = 0.944) (Supplementary Table S2). 

### 3.4. Factors Independently Associated with CIMT 

Finally, we carried out a multivariate linear regression analysis seeking to identify any factors associated with CIMT in the sample of patients, adjusting the model for hypertension, low-density lipoprotein (LDL) level, diabetes mellitus and smoking, given known CVRFs, and other treatments such as methotrexate, glucocorticoids or statins. Our results show that sex male, older age and having a greater cumulative ESR (but not ESR at the time of the visit) were independently associated with a larger CIMT, while patients on TNFα inhibitors had a CIMT 0.080 mm smaller than those on other treatments. Other treatments were not related with having a lower CIMT (Table 3).

## 4. Discussion

The elevated cardiovascular risk in patients with RA is, to a great extent, due to the systemic inflammatory nature of their condition [16,17]. Given the relationship between inflammatory response and atherosclerosis, the management of inflammation is currently considered an effective strategy for modifying CVRFs and thereby preventing cardiovascular events [18,25]. Few studies have assessed the effect of biological therapies now available on subclinical atherosclerosis in patients with RA, most having focused on evaluating conventional DMARDs and very few considering biological therapies [20,21]. In our study, we have included all the biological and targeted therapies currently available to treat RA and assessed their association with subclinical atherosclerosis using carotid ultrasound. Our results indicate that patients treated with TNFα inhibitors, but not other biological therapies, have the smallest CIMT, and hence, a lower rate of subclinical atherosclerosis.

Regarding traditional CVRFs, our patients had higher rates of hypertension and dyslipidaemia than controls, as in previous studies [26], the patients also having a higher BMI than controls. Nonetheless, we did not observe differences in blood lipid levels, despite all the patients with a diagnosis of dyslipidaemia being on statins. In addition, we did not find an association between statins intake and CIMT in the RA group. Other studies have shown that only intensive cholesterol-lowering therapy regressed the carotid atherosclerosis over one year, which is not our case [27]. We observed higher levels of homocysteine among patients and a linear correlation between these levels and CIMT, but this correlation was weak and this variable was not included in the multivariate analysis. Homocysteine is known to be an independent risk factor for cardiovascular events, given its involvement in endothelial dysfunction and LDL oxidation as well as its ability to promote the proliferation of vascular smooth muscle cells [28]. Previous studies have reported high levels of this amino acid in patients with RA [29,30].

Regarding subclinical atherosclerosis as measured by carotid ultrasound, we found that patients were more likely to have atheromatous plaque than controls, consistent with previous studies [31,32]. On the other hand, while there was a trend towards a larger CIMT, the differences did not reach statistical significance. This finding has also been reported in previous studies [33] and may be due to the relatively low inflammatory activity in our patients, which might have had a beneficial effect on CIMT. Other research has demonstrated that effective control of the disease may abrogate any specific effect on RA on atherosclerosis progression [34].

The rates of RF and anti-CCP positivity and radiological erosions were similar to those reported in other Spanish series of patients with RA [35,36]. On the other hand, we did not find significant associations between these factors and the CIMT, which have been observed in smaller cohorts [33,37]. Regarding acute-phase reactants, as in other research [38], a correlation was observed between ESR and CIMT. In our study, we included a parameter corresponding to the mean of all measurements of ESR taken in the previous 2 years, believing that this might provide more information than an isolated measurement, since cardiovascular risk is ultimately the result of a cumulative inflammatory process, together with other CVRFs, and not of a single factor, in this case, inflammation at one particular time [39,40]. And notably, we demonstrated a linear correlation between CIMT and this cumulative ESR, but not with ESR as measured at the time of the visit. Further, this finding was confirmed in the final multivariate linear analysis.

In relation to the different biological therapies, unlike in previous smaller studies, we also included patients on IL-6 inhibitors and jakinibs [21]. We only found CIMT to be smaller in patients on TNFα inhibitors or jakinibs compared to values in those on other therapies. Comparing patients on jakinibs with those on other treatments, we found no differences in terms of CVRFs, blood lipid levels or disease activity, which could lead us to suppose that the drug itself is responsible for the reduction in CIMT. Nonetheless, the number of patients on jakinibs was relatively small and therefore this finding should be interpreted with caution. Indeed, it was not confirmed in the multivariate analysis. Further, there may be bias in clinical practice when we initiate this type of therapy due to concerns regarding some jakinibs and cardiovascular events [41,42], and that given this, we may be selecting patients who have fewer CVRFs and are younger for this treatment. A study of 46 patients prospectively assessed the effect of treatment with tofacitinib and methotrexate on CIMT and observed a significant reduction in 12 patients [43].

Regarding treatment with rituximab and abatacept, we did not find differences in CIMT, but we did find that patients on these drugs were more likely than those on other therapies to have atheromatous plaque. Few studies have assessed this factor; among those that have, a 6-month follow-up study in 55 patients on treatment with rituximab only demonstrated an 11% narrower CIMT in patients in whom the disease was under control with this therapy ([44] and a more recent study including only 13 patients on rituximab did not find more atheromatous plaque in these patients [21]. Some studies in mice suggest a protective effect of rituximab on atherosclerosis, in that depletion of B cells would have an effect on proatherogenic anti-oxidized LDL antibodies, as bonding between oxidated LDL and anti-oxLDL IgG would lead to the production of immune complexes that bind to macrophages, forming foam cells, in turn resulting in the release of proinflammatory cytokines and contributing to the formation of atheromatous plaque ([45]. As for abatacept, we have found only one study on this topic and the authors concluded that using this therapy, CIMT could be kept stable [46]. 

Although the number of patients on tocilizumab was similar to that on TNFα inhibitors, we did not find differences in CIMT or atheromatous plaque with this treatment. Previous research, specifically a study by our research group, has suggested a beneficial role of tocilizumab on cardiovascular risk, reducing the pro-atherothrombotic profile in patients with RA through restoration of endothelial function, reduction in oxidative stress, inhibition of the prothrombotic and inflammatory characteristics of monocytes and abridged NETosis generation [47]. To our knowledge, however, no studies have previously explored subclinical atherosclerosis using carotid ultrasound in these patients.

Regarding treatment with TNFα inhibitors, our results show that patients on this treatment have smaller CIMT than patients on other therapies in this cohort of patients, regardless of traditional CVRFs and the inflammatory load of the disease as measured using cumulative ESR over the previous 24 months, though we did not find such differences in terms of atheromatous plaque. This might be explained by the formation of plaque being a more advanced stage in the process of atherosclerosis than an increase in CIMT. Such an increase may be also the result of subclinical vasculitis and/or carotid artery wall hypertrophy, as previously demonstrated in patients with RA [48], though it has also been demonstrated that CIMT without atheromatous plaque is still a significant marker of higher cardiovascular risk and development of plaque [14,49,50].

Findings concerning the effect of TNFα inhibitors on the progression of subclinical atherosclerosis in patients with RA are inconsistent [51]. Some studies have reported a progression of CIMT in patients with high disease activity [52], but most have indicated that with this treatment, CIMT does not significantly change [53,54] or even decreases [55]. In our study, exploring potential differences between patients on TNFα inhibitors and those on other treatments, we did not find differences in terms of inflammatory activity, acute phase reactants or CVRFs and, hence, it may be possible that the specific characteristics of this type of drug are responsible for the smaller CIMTs observed, although the exact mechanism by which TNFα inhibitors might reduce cardiovascular risk remains unclear.

One of the main limitations of our study is its cross-sectional nature, and therefore, we are unable to infer that treatment with TNFα inhibitors was the cause of the smaller CIMT observed. Further, to obtain more robust results, there is a need to study larger samples of patients on each type of biological therapy. Another limitation may be represented by the inclusion of patients with a previous history of cardiovascular events, which might influence the results. However, only four patients in the RA group had a previous history of a cardiovascular event, without significant difference with the control group. On the other hand, in this study, we included all the biological therapies currently available for RA and recruited a larger number of patients than other studies, and we have found promising results in the case of TNFα inhibitors.

To summarise, our study indicates that the use of TNFα inhibitors may have a protective effect against subclinical atherosclerosis in patients with RA, given that patients on this biologic have a smaller CIMT than patients on other disease-modifying antirheumatic drugs. Nonetheless, these results should be confirmed in prospective studies with larger sample sizes.

## Figures and Tables

**Table 1:** Baseline demographic and epidemiological characteristics, comorbidities, lab results and carotid ultrasound findings in patients and controls.

Variable	Patients (*n* = 146)	Controls (*n* = 30)	*p*-Value
**Baseline demographic and epidemiological characteristics**			
Age in years, mean (SD)	55.8 (11.5)	52.4 (7.9)	0.062
Sex (women), *n* (%)	112 (76.7)	20 (66.6)	0.247
Smoking status			0.211
Never smoker, *n* (%)	86 (61.9)	17 (56.6)	
Ex-smoker, *n* (%)	22 (15.8)	6 (22.2)	
Active smoker, *n* (%)	31 (22.3)	2 (7.4)	
High blood pressure, *n* (%)	43 (29.5)	4 (13.3)	0.069
Diabetes mellitus, *n* (%)	4 (2.7)	1 (4)	0.729
Heart disease, *n* (%)	4 (2.7)	0	0.988
Dyslipaemia, *n* (%)	46 (31.5)	1 (4)	**0.004**
Body mass index, mean (SD)	27.3 (5.5)	24.4 (3.4)	**0.014**
**Lab results**			
Uric acid (mg/dl), mean (SD)	4.5 (1.3)	4.9 (1.1)	0.321
Total cholesterol (mg/dl), mean (SD)	198.5 (37.6)	206 (26.2)	0.106
LDL cholesterol (mg/dl), mean (SD)	119.2 (30.2)	126.8 (21.7)	0.134
HDL cholesterol (mg/dl), mean (SD)	59.6 (17.8)	59.4 (17.2)	0.553
Triglycerides (mg/dl), mean (SD)	102 (47.3)	100 (45.8)	0.630
Apolipoprotein A1 (mg/dl), mean (SD)	150 (32.5)	158 (31.0)	0.226
Apolipoprotein B (mg/dl), mean (SD)	86.7 (22.1)	84.9 (15.8)	0.634
ApoB/ApoA1, mean (SD)	0.61 (0.23)	0.55 (0.14)	0.156
Homocysteine (mg/L), mean (SD)	2.9 (3.6)	1.9 (0.5)	**0.022**
ESR (mm/h) at visit, mean (SD)	16.8 (14.4)	12.3 (8.2)	**0.016**
CRP (mg/dl) at visit, mean (SD)	8.8 (13.5)	2.2 (2.1)	**<0.001**
2-year mean CRP, mean (SD)	9.7 (11.5)	-	-
2-year mean ESR, mean (SD)	17.5 (12.1)	-	-
**Characteristics of the disease**			
Disease duration of RA (years), mean (SD)	8.8 (8.1)	-	-
Erosions, *n* (%)	53 (36.6)	-	-
Rheumatoid nodules, *n* (%)	9 (6.2)	-	-
Rheumatoid factor positive, *n* (%)	120 (82.2)	0	**<0.001**
Anti-CCP positive, *n* (%)	124 (84.9)	0	**<0.001**
Anti-CCP level, mean (SD)	315.9 (520.2)	-	-
Rheumatoid factor level, mean (SD)	114 (4.1)	-	-
Cumulative DAS28, mean (SD)	3 (1.22)	-	-
Remission-low activity, *n* (%)	75 (58.1)	-	-
Cumulative CDAI, mean (SD)	10.9 (7.3)	-	-
Cumulative SDAI, mean (SD)	11.8 (7.6)	-	-
HAQ at visit, mean (SD)	0.79 (0.68)	-	-
**Treatment at the time of the study visit**			
Methotrexate as monotherapy, *n* (%)	26 (17.8)	-	-
MTX + HCQ, *n* (%)	26 (17.8)	-	-
Another DMARD combination, *n* (%)	16 (10.9)	-	-
bDMARD, *n* (%) bDMARD treatment durations (years), mean (SD)	68 (46.57) 3.6 (3.9)	- -	- -
TNFα inhibitors, *n* (%)	26 (17.8)	-	-
Abatacept, *n* (%)	9 (6.2)	-	-
IL-6 inhibitors, *n* (%)	22 (15.1)	-	-
Rituximab, *n* (%)	11 (7.5)	-	-
Janus kinase inhibitors, *n* (%)	8 (5.5)	-	-
csDMARDs + bDMARDs, *n* (%)	56 (38,3)	-	-
Glucocorticoids, *n* (%)	83 (56,8)	-	-
Glucocorticoid dose at time of visit (mg), mean (SD) Statins at time of visit, *n* (%)	3.7 (4.2) 33 (22.6)	- 0 (0)	- **0.002**
**Carotid ultrasound**			
Atheromatous plaque, *n* (%)	49 (33.8)	3 (12.5)	**0.036**
Bilateral distribution of plaque, *n* (%)	16 (11.3)	1 (3.3)	0.239
CIMT, mean (SD)	0.64 (0.18)	0.61 (0.13)	0.444

Abbreviations: LDL: low-density lipoprotein; HDL: high-density lipoprotein; ApoB: apolipoprotein B; ApA1: apolipoprotein A1; CRP: C-reactive protein; ESR: erythrocyte sedimentation rate; anti-CCP: anti-cyclic citrullinated peptide, DAS28: 28-joint Disease Activity Score; CDAI: Clinical Disease Activity Index; SDAI: Simplified Disease Activity Index; HAQ Health Assessment Questionnaire; bDMARD: biological disease-modifying antirheumatic drug; csDMARD: synthetic disease-modifying antirheumatic drug; TNF: tumour necrosis factor; IL-6: interleukin 6; CIMT: carotid intima-media thickness.

**Table 2:** Relationship between carotid ultrasound findings and various therapies studied (biologics and targeted synthetic disease-modifying antirheumatic drugs).

Treatment	Carotid Intima-Media Thickness		Atheromatous Plaque	
	Mean in mm (SD)	*p*-Value	*n* (%)	*p*-Value
TNFα inhibitor (+) vs. TNFα inhibitor (−)	O.58 (0.10) vs. 0.65 (0.19)	**0.013**	6 (23.1) vs. 43 (35.8)	0.202
IL-6 inhibitor (+) vs. IL-6 inhibitor (−)	0.60 (0.09) vs. 0.64 (0.19)	0.165	4 (18.2) vs. 45 (35.7)	0.093
Rituximab (+) vs. Rituximab (−)	0.68 (0.17) vs. 0.63 (0.18)	0.358	7 (63.7) vs. 42 (31.1)	**0.029**
Abatacept (+) vs. Abatacept (−)	0.60 (0.09) vs. 0.64 (0.19)	0.408	6 (66.7) vs. 43 (31.6)	**0.031**
Jakinibs (+) vs. Jakinibs (−)	0.52 (0.02) vs. 0.64 (0.18)	**<0.001**	1 (12.5) vs. 48 (34.8)	0.190

Abbreviations: IL-6: Interleukin 6; TNF, tumour necrosis factor; MTX: methotrexate.

**Table 3:** Multivariate linear regression model with the sample of patients.

Dependent Variables	Predictor	β Coefficient	95% CI for B	*p*-Value
CIMT	Sex (male)	0.066	0.0008- 0.130	**0.047**
	Age (years)	0.006	0.002–0.008	**<0.001**
	Cumulative ESR	0.003	0.0007–0.005	**0.011**
	Hypertension	0.016	(−0.05–0.085)	0.645
	LDL levels	0.0009	(−0.0001–0.001)	**0.027**
	Diabetes Mellitus	0.121	(−0.041–0.283)	0.141
	Smoking	−0.008	(−0.063–0.045)	0.742
	Disease duration (years)	−0.002	(−0.005–0.001)	0.168
	Methotrexate	−0.004	(−0.064–0.055)	0.882
	bDMARDs (anti-TNFα)	−0.080	(−0.153)–(−0.007)	**0.031**
	bDMARDs (abatacept)	−0.051	(−0.159–0.057)	0.353
	bDMARDs (rituximab)	−0.039	(−0.141–0.063)	0.452
	bDMARDs (anti-IL6)	−0.003	(−0.087–0.081)	0.943
	Jakinibs	−0.063	(−0.087–0.081)	0.291
	Glucocorticoids	−0.024	(−0.078–0.030)	0.381
	Statins	0.022	(−0.036–0.081)	0.455
	Atheromatosus plaques	0.049	(−0.019–0.117)	0.156

R^2^ 0.369. Abbreviations: CIMT: carotid intima-media thickness; LDL: low-density lipoprotein.

## Data Availability

Data presented in this study are available on request from the corresponding author.

## Supplementary Materials

The following are available online at https://www.mdpi.com/article/10.3390/diagnostics12010064/s1, Table S1: Differences between patients on TNFα inhibitors and those on other therapies. Table S2: Differences between patients on Janus kinase inhibitors and those on other therapies.
