# Supplementary material for: Association between Carotid Intima-Media Thickness and the Use of Biological or Small Molecule Therapies in Patients with Rheumatoid Arthritis
Source: Diagnostics (Basel). 2021 Dec 28;12(1):64. doi: 10.3390/diagnostics12010064 (PMC8775122; doi:10.3390/diagnostics12010064)
Supplement: Supplementary file 1 [file diagnostics-12-00064-s001.zip › diagnostics-1511926-supplementary.pdf]

Supplementary Table S1. Differences between patients on TNFα inhibitors and those on other therapies

Variable	TNFα inhibitors n= 26	Other therapies n=120	*p-value*
**Baseline demographic and epidemiological characteristics**			
Age in years, mean (SD)	54.9 (9.4)	55.9 (12.05)	0.647
Sex (women), n (%)	19 (73.1)	93 (77.5)	0.628
Smoking status			0.488
Never smoker, n (%)	17 (65.4)	69 (57.5)	-
Ex-smoker, n (%)	2 (7.7)	20 (16.6)	-
Active smoker n (%)	5 (19.2)	26 (21.6)	-
Body mass index (Kg/m^2), mean (SD)	30.3 (7.9)	26.6 (4.7)	**0.040**
**Comorbidities**			
Hypertension, n (%)	3 (11.5)	40 (33.3)	**0.027**
Diabetes mellitus, n (%)	1 (3.8)	3 (2.5)	0.703
Heart disease, n (%)	0	2 (1.7)	0.498
Dyslipidaemia, n (%)	5 (19.2)	41 (34.1)	0.137
**Lab results**			
Uric acid (mg/dl), mean (SD)	4.9 (1.4)	4.4 (1.3)	0.226
Total cholesterol (mg/dl), mean (SD)	208.5 (36.7)	195.7 (37.4)	0.119
LDL cholesterol (mg/dl), mean (SD)	126.8 (32.9)	117.1 (29.5)	0.176
HDL cholesterol (mg/dl), mean (SD)	59.8 (13)	59.4 (18.8)	0.891
Triglycerides (mg/dl), mean (SD)	105 (46.2)	102.1 (47.7)	0.733
Apolipoprotein A1 (mg/dl), mean (SD)	151 (29.8)	150.2 (33.1)	0.906
Apolipoprotein B (mg/dl), mean (SD)	98.5 (26.5)	83.7 (20.03)	**0.016**
ApoB/ApoA1, mean (SD)	0.7 (0.36)	0.57 (0.2)	0.105
Homocysteine (mg/L), mean (SD)	1.9 (0.55)	3.3 (4.1)	**0.018**
ESR (mm/h) at visit, mean (SD)	16.8 (12.7)	16.8 (14.8)	0.992
2-year mean ESR, mean (SD)	18.02 (10.8)	17.4 (12.4)	0.824
CRP (mg/dl) at visit, mean (SD)	8.41 (7.6)	8.9 (14.5)	0.781
2-year mean CRP, mean (SD)	9.2 (8.9)	9.8 (12)	0.782
**Characteristics of the disease**			
Disease duration of RA (years), mean (SD)	9.9 (7.2)	8.6 (8.3)	0.419
Rheumatoid factor positive, n (%)	19 (73.1)	105 (87.5)	**0.035**
Anti-CCP positive, n (%)	21 (80.7)	103 (85.8)	0.512
Anti-CCP level, mean (SD)	310.1 (807)	317 (439)	0.966
RF level, mean (SD)	102.8 (105)	116.4 (193.2)	0.627
Cumulative DAS28, mean (SD)	3.21 (1.3)	2.9 (1.2)	0.333
Remission-low activity, n (%)	13 (50)	62 (51.6)	0.346
Cumulative CDAI, mean (SD)	13.3 (8.8)	10.3 (6.8)	0.119
Cumulative SDAI, mean (SD)	13.9 (8.8)	11.2 (7.3)	0.151
HAQ at visit, mean (SD)	1.05 (0.29)	0.73 (0.7)	0.059
**Carotid ultrasound**			
CIMT, mean (SD)	0.58 (0.1)	0.65 (0.2)	**0.013**
Atheromatous plaque, n (%)	6 (23.1)	42 (35.8)	0.202
Bilateral distribution of plaque, n (%)	1 (3.8)	15 (12.5)	0.185

Abbreviations: LDL: low-density lipoprotein; HDL: high-density lipoprotein; ApoB: apolipoprotein B; ApA1: apolipoprotein A1; CRP: C-reactive protein; ESR: erythrocyte sedimentation rate; anti-CCP: anti-cyclic citrullinated peptide, DAS28: 28-joint Disease Activity Score; CDAI: Clinical Disease Activity Index; SDAI: Simplified Disease Activity Index; HAQ Health Assessment Questionnaire; bDMARD: biological disease-modifying antirheumatic drug; tsDMARD: targeted synthetic disease-modifying antirheumatic drug; TNF: tumour necrosis factor; IL-6: interleukin 6; CIMT: carotid intima-media thickness.

Supplementary Table S2. Differences between patients on Janus kinase inhibitors and those on other therapies

Variable	Jakinibs n= 8	Other therapies n=138	*p-value*
**Baseline demographic and epidemiological characteristics**			
Age in years, mean (SD)	52.2 (12.8)	55.9 (12.05)	0.442
Sex (women), n (%)	7 (87.5)	93 (77.5)	0.457
Smoking status			0.447
Never smoker, n (%)	6 (75)	69 (57.5)	-
Ex-smoker, n (%)	0 (0)	20 (16.6)	-
Active smoker, n (%)	2 (25)	26 (21.6)	-
Body mass index (kg/m^2), mean (SD)	25.1 (3.9)	26.6 (4.7)	0.154
**Comorbidities**			
Hypertension, n (%)	2 (25)	40 (33.3)	0.776
Diabetes mellitus, n (%)	0 (0)	3 (2.5)	0.625
Heart disease, n (%)	1 (12.5)	2 (1.7)	0.081
Dyslipidaemia, n (%)	3 (37.5)	41 (34.1)	0.707
**Lab results**			
Uric acid (mg/dl), mean (SD)	3.8 (1.1)	4.4 (1.3)	0.065
Total cholesterol (mg/dl), mean (SD)	180.6 (58.6)	195.7 (37.4)	0.402
LDL cholesterol (mg/dl), mean (SD)	117.7 (36.5)	117.1 (29.5)	0.920
HDL cholesterol (mg/dl), mean (SD)	59.7 (16.7)	59.4 (18.8)	0.971
Triglycerides (mg/dl), mean (SD)	91.5 (27.8)	102.1 (47.7)	0.288
Apolipoprotein A1 (mg/dl), mean (SD)	152 (29.5)	150.2 (33.1)	0.886
Apolipoprotein B (mg/dl), mean (SD)	79.5 (35.7)	83.7 (20.03)	0.595
ApoB/ApoA1, mean (SD)	0.6 (0.3)	0.57 (0.2)	0.716
Homocysteine (mg/L), mean (SD)	1.8 (0.4)	3.3 (4.1)	0.060
ESR (mm/h) at visit, mean (SD)	19.6 (14.8)	16.8 (14.8)	0.594
2-year mean ESR, mean (SD)	24.1 (20.1)	17.4 (12.4)	0.367
CRP (mg/dl) at visit, mean (SD)	3.8 (3.9)	8.9 (14.5)	**0.008**
2-year mean CRP, mean (SD)	11.1 (14.9)	9.8 (12)	0.796
**Characteristics of the disease**			
Disease duration of RA (years), mean (SD)	8.4(8.05)	8.6 (8.3)	**0.004**
Rheumatoid factor positive, n (%)	8 (100)	105 (87.5)	0.891
Anti-CPP positive, n (%)	8 (100)	103 (85.8)	0.220
Anti-CPC level, mean (SD)	256 (152)	317 (439)	0.406
RF level, mean (DE)	115.9 (126.7)	116.4 (193.2)	0.969
Cumulative DAS28, mean (SD)	3.1 (0.9)	2.9 (1.2)	0.844
Remission-low activity, n (%)	6 (75)	62 (51.6)	0.407
Cumulative CDAI, mean (SD)	9.8 (6.02)	10.3 (6.8)	0.608
Cumulative SDAI, mean (SD)	10.7 (6.4)	11.2 (7.3)	0.608
HAQ at visit, mean (SD)	0.68 (0.6)	0.73 (0.7)	0.851
**Carotid ultrasound**			
CIMT, mean (SD)	0.52 (0.02)	0.64 (0.18)	**<0.001**
Atheromatous plaque, n (%)	1 (12.5)	48 (34.8)	0.190
Bilateral distribution of plaques, n (%)	0 (0)	16 (11.6)	0.299

Abbreviations: jakinibs: Janus kinase inhibitors; LDL: low-density lipoprotein; HDL: high-density lipoprotein; ApoB: apolipoprotein B; ApA1: apolipoprotein A1; CRP: C-reactive protein; ESR: erythrocyte sedimentation rate; anti-CCP: anti-cyclic citrullinated peptide, DAS28: 28-joint Disease Activity Score; CDAI: Clinical Disease Activity Index; SDAI: Simplified Disease Activity Index; HAQ Health Assessment Questionnaire; bDMARD: biological disease-modifying antirheumatic drug; tsDMARD: targeted synthetic disease-modifying antirheumatic drug; TNF: tumour necrosis factor; IL-6: interleukin 6; CIMT: carotid intima-media thickness.
